# Swine Dendritic Cell Response to Porcine Reproductive and Respiratory Syndrome Virus: An Update

**DOI:** 10.3389/fimmu.2021.712109

**Published:** 2021-07-28

**Authors:** Jesús Hernández, Yanli Li, Enric Mateu

**Affiliations:** ^1^Laboratorio de Inmunología, Centro de Investigación en Alimentación y Desarrollo, Hermosillo, Mexico; ^2^Departament de Sanitat i Anatomia Animals, Facultat de Veterinària, Universitat Autònoma de Barcelona, Cerdanyola del Vallès, Spain; ^3^IRTA, Centre de Recerca en Sanitat Animal (CReSA, IRTA-UAB), Cerdanyola del Vallès, Spain

**Keywords:** PRRSV, dendritic cell, cDC1, cCD2, pDCs, bmDCs, moDCs

## Abstract

Dendritic cells (DCs) are the most potent antigen-presenting cells, unique to initiate and coordinate the adaptive immune response. In pigs, conventional DCs (cDCs), plasmacytoid DCs (pDCs), and monocyte-derived DCs (moDCs) have been described in blood and tissues. Different pathogens, such as viruses, could infect these cells, and in some cases, compromise their response. The understanding of the interaction between DCs and viruses is critical to comprehend viral immunopathological responses. Porcine reproductive and respiratory syndrome virus (PRRSV) is the most important respiratory pathogen in the global pig population. Different reports support the notion that PRRSV modulates pig immune response in addition to their genetic and antigenic variability. The interaction of PRRSV with DCs is a mostly unexplored area with conflicting results and lots of uncertainties. Among the scarce certainties, cDCs and pDCs are refractory to PRRSV infection in contrast to moDCs. Additionally, response of DCs to PRRSV can be different depending on the type of DCs and maybe is related to the virulence of the viral isolate. The precise impact of this virus-DC interaction upon the development of the specific immune response is not fully elucidated. The present review briefly summarizes and discusses the previous studies on the interaction of *in vitro* derived bone marrow (bm)- and moDCs, and *in vivo* isolated cDCs, pDCs, and moDCs with PRRSV1 and 2.

## Introduction

Porcine reproductive and respiratory syndrome (PRRS) is one of the most important diseases affecting the swine industry worldwide. It causes reproductive failure in sows and respiratory distress, growth reduction, and high mortality in young pigs (1). Economically, it is important for the affected farms; for example, losses associated with the disease were estimated at 644 million USD annually for US farms (2). The etiological agents of PRRS are two enveloped positive-sense RNA viruses designated as porcine reproductive and respiratory syndrome virus (PRRSV) 1 and 2. Both PRRSV viruses belong to the family *Arteriviridae*, genus Betaarterivirus (3). PRRSV genome is about 15 kb in length containing at least ten open reading frames (ORFs) flanked by two untranslated regions at 5’ and 3’ ends. The ORFs1a and 1b encompass about 80% of the viral genome and encode two polyproteins that are cleaved into 14 non-structural proteins (nsp1-nsp14). ORF2a, ORF2b, ORFs 3 - 7, and ORF5a genes encode eight structural proteins: GP2, E, GP3, GP4, GP5, M, N, and ORF5a protein, respectively (4).

Non-structural proteins are essential for viral replication, are recognized by the immune system, and have been involved in regulating of the immune response (5). Structural proteins have been implicated in the modulation of host innate immune response and the induction of antigen-specific B- and T-cell responses [Reviewed by Loving et al. (6)]. After infection, pigs rapidly develop antibodies, but those early antibodies are devoid of neutralizing capacities. Antibodies with neutralizing activity appear later, usually after the fourth week of infection (7). These antibodies are mainly directed against GP2, GP3, GP4, and GP5, proteins interacting with CD163 (GP2-GP4) and porcine sialoadhesin (Siglec-1, GP5) which are the main receptors mediating PRRSV entry into the target cells (8). The induction of PRRSV-specific IFN-γ-secreting cells (IFN-γ-SC) also occurs relatively late in the course of the infection (7, 9) which is considered an indicator of the development of cell-mediated immunity. It has been proved that both structural and non-structural viral proteins induce IFN-γ-SC (10–12).

The delayed development of neutralizing antibodies and late production of cell-mediated responses result in prolonged viremia and persistence of the virus in lymphoid tissues for several weeks. Different hypotheses have been proposed to explain this unusual immune response, many of them suggesting that the interaction of the virus with dendritic cells may be the main cause. This review discusses the response and modulatory effects of PRRVS on monocyte- (mo-), bone-marrow- (bm) derived DCs, conventional DCs (cDCs), and plasmacytoid DCs (pDCs).

## Dendritic Cells

DCs are professional antigen-presenting cells. They sense the presence of invading antigens, then migrate to the lymph nodes, particularly the T cell-rich zones, to trigger naïve lymphocytes into distinct classes of effector cells, finally initiate the T cell-mediated immunity (13, 14). There are mainly two types of DCs: plasmacytoid DCs (pDCs) and myeloid conventional or classical DCs (cDCs). cDCs are further divided into two subtypes or subsets: cDC1 and cDC2 (15). pDCs are responsible for producing interferon-alpha (IFN-α) in response to viral infections, but are less efficient at T-cell priming than cDCs (16). In contrast, cDCs are the most professional antigen-presenting cells. The cDC1 subset is thought to drive naïve T cells into Th1 cells and cross-present antigens to CD8 T cells (17). The cDC2 subtype stimulates CD4 T cells to promote Th2 and Th17 cells (18, 19).

The cDC1 and cDC2 subtypes have been characterized in several species, including swine (20). Using common markers shared by humans, macaques, and mice, Guilliams et al. (21) identified the cDC1 subtype as XCR1^hi^CADM1^hi^CD172a^low^IRF8^hi^IRF4^lo^, and the cDC2 subtype as XCR1^-^CD172a^hi^IRF8^lo^IRF4^hi^ (21). In swine, significant contributions have been made in the last years, allowing identifying cDCs, and the cDC1 and cDC2 subtypes from blood (22, 23), lymph nodes (24, 25), skin (26), and lung (27). In all these reports, cDC1 and cDC2 have been characterized, although in some cases the labeling strategy was different. These differences will not be further discussed in this review.

Monocytes may capture antigens, present them in the histocompatibility complex and differentiate into the so-called moDCs in peripheral non-lymphoid organs, particularly under inflammatory conditions (28). *In vivo* generated moDCs may possess the cross-presentation ability and are able to transfer antigens to DCs present in lymph nodes, thus being important in developing an immune response to pathogens [see (28) for a review on the role of moDC]. It is worth mentioning that moDCs were increased in lungs of PRRSV-infected animals (29). moDCs and bmDCs have been extensively used in *in vitro* studies as well to analyze the interaction between PRRSV and DCs. Most of those reports used a similar strategy consisting of culturing monocytes or bone-marrow cells with GM-CSF with or without the addition of IL-4 to differentiate DCs. These protocols will neither be discussed, and only specific annotations will be done if necessary.

## Monocyte- and Bone Marrow-Derived DCs Response to PRRSV

For many years, the difficulty for having *ex vivo* cDC or for deriving them from precursors render moDCs and bmDCs a useful method to evaluate the response of DCs to PRRSV. Both moDCs and bmDCs are heterogeneous cell populations that include subsets susceptible to infection and replication, allowing to evaluate the effects of infection and co-infection with other pathogens. Most studies indicated that both PRRSV1 and 2 could enter and replicate in moDCs (30–34) and bmDCs (35) with one study indicating lower susceptibility of moDCs than bmDCs (36). The viral yield after infection may differ in bmDCs and moDCs (37) and not all strains would have the same replication rates in moDCs as those obtained in monocyte-derived macrophages (38). These differences could be explained by the low proportion of CD163^pos^ (and to a less extent by the low expression of Siglec-1/CD169 in moDCs) or participation of other receptors (38).

One of the main focuses when studying the interaction of PRRSV with moDCs and bmDCs is the cytokine patterns after exposure to PRRSV. IL-10 is a potent anti-inflammatory cytokine with a broad range of effects on the immune response against viral infections. It is produced by a variety of cells, including DCs. It has been reported that pathogens inducing IL-10 at early phases of the immune response could compromise the resolution of infection, and in some cases, lead to persistent infection or promote maintenance of persistent infection (39).

Production of IL-10 by PRRSV-infected DCs remains controversial. Some authors reported significant production after infection of moDCs (31, 32, 40) or bmDCs (35, 41, 42), while others reported either a non-significant induction of this cytokine (30, 43) or that IL-10 production was strain-dependent (44, 45). In other cases, IL-10 was produced only when moDCs were co-infected with PRRSV and other agents such as PCV2 but not by PRRSV alone (46). These studies suggest that IL-10 production may depend on several factors including, but maybe not limited to, the maturation stage of DCs and the PRRSV strain used. Also, it could be enhanced by coinfection or superinfection with other pathogens. *In vivo* production of IL-10 has been more consistently reported, suggesting that in addition to DCs, other cells, such as macrophages, in the lung or lymphoid tissues, could be important sources of this cytokine during PRRSV infection (47). Liu et al. (36) proposed that IL-10 production was predominant in PRRSV-infected moDCs, suppressing the production of Th1 cytokines, such as IL-12, and finally regulating the polarization of Th1/Th2.

It has been reported that several pathogens can induce T regulatory cells (Tregs) [reviewed by Maizels and Smith (48)]. Tregs are important sources of regulatory cytokines (IL-10 or TGF-β) which can suppress or delay naïve T-cell priming and proliferation, (49) thus impairing the protective responses. Two main types of Tregs, natural (n) and induced (i), have been recognized (50). nTregs arise in the thymus, while iTregs arise in the periphery following CD4^+^ T-cell activation in the presence of TGF-β or upon stimulation with IL-2 or exposure to some pathogens (50).

It has been suggested that moDCs infected by PRRSV were able to induce the development of Tregs with suppressive activity *in vitro* (51, 52) and *in vivo* in the blood (52), lymphoid tissues (53), and lung (54). When the mononuclear cells isolated from blood and lung were restimulated *ex vivo* with PRRSV, TGF-β and IL-10 were produced, suggesting that Tregs might play an essential role in PRRSV immunopathogenesis. These Tregs seem to be induced mainly by PRRSV2 since studies using PRRSV1 failed to demonstrate the development of Tregs (33). The higher virulence of PRRSV2 isolates has been used to explain these differences (33). Other groups using moDCs infected with PRRSV1 or PRRV2 did not observe induction or proliferation of Tregs (55). Moreover, Bordet et al. (29) did not observe IL-10 or TGF-β induced by highly virulent PRRSV1.3 Lena although Tregs were not assessed (29). Also, Li and Mateu (56) did not observe a significant proliferation of Tregs induced by *in vitro* derived allogeneic PRRSV1-stimulated cDCs (56). Given the diversity of PRRSV, further studies are required to determine whether PRRSV1 differed from PRRSV2 in inducing Tregs. Interestingly, heat-inactivated PRRSV or pre-treated DCs with IFN-α reduced the expansion of Tregs, indicating that viral replication is probably necessary for Treg induction (51).

The viral nucleocapsid protein (N) was presumed to be an inducer of Tregs as transfection of moDCs with the N protein induced an increase of Tregs and a comparable level of IL-10 in PBMCs as the live virus-stimulated moDCs (57). Moreover, neutralization of IL-10 in the cultures of transfected moDCs reduced the number of Tregs, reinforcing the notion that IL-10 induction by N was responsible for the observed effect (58). However, in other models such as the co-infection with PCV2, the predominant regulatory cytokine was TGF-β although its level could not be correlated with the number of Tregs (46).

The discussion above suggests that PRRSV2 can induce Tregs through moDCs-mediated antigen presentation, but it remains unknown whether *bona fide* DCs could also induce Tregs since they are not susceptible (discussed below). It is also unclear how Tregs contribute to the immunopathogenesis of PRRSV, or if they only represent a homeostatic response to inflammatory events.

In addition to the induction of Tregs, Nedumpun et al. showed that PRRSV-infected moDCs induced the expression and production of IL-1Ra, which is a natural suppressor of the inflammatory response (59, 60). Chen et al. (61) demonstrated that the viral N protein and nsp10 enhanced the production of CD83, an inhibitor of DC-mediated T-cell proliferation (62), *via* activation of NF-KB and Sp1 signaling pathways (61).

The effects of co-infection or super-infection on the functionality of DCs have been evaluated with different *in vitro* models. Concomitant infections frequently occur in the field and PRRSV infection can have an impact on the response of DCs against other pathogens of pigs. For example, exposure to PRRSV impaired the capacity of bmDCs in ingesting *Streptococcus suis* (*S. suis*) although the killing of phagocytized bacteria was not affected. In the same study, PRRSV and *S. suis* showed a synergistic interaction on the expression of chemokines and pro-inflammatory cytokines (37). In another study, coinfection of moDCs with PRRSV and PCV2 resulted in an increased expression of PD-L1 (63), a molecule associated with negative regulation of T-cell stimulation (64). However, this effect was dependent on the PRRSV strain used. These results suggested that moDCs co-infected with PRRSV and PCV2 could suppress the immune response and favor PCAVD pathogenesis (63).

## Response of Conventional DCs Against PRRSV

One crucial question regarding PRRSV immunology is whether PRRSV infects, replicates, and modulates the response of cDCs. According to the *in vitro* models using moDCs and bmDCs, the immunopathology of PRRSV is due, at least partially, to the infection of DCs. However, the few studies published dealing with *bona fide* cDCs isolated from pigs or used *ex vivo* showed a different picture. For example, Loving et al. (65), analyzing lung cDCs, concluded that PRRSV did not replicate in them (65). Using a similar strategy, Proll et al. (66) reported that DCs were susceptible to PRRSV1 infection and explained the discrepancy of results as the type (PRRSV1 *vs.* PRRSV2) of virus used (66). Another explanation is the approach to get the DCs in both studies. The authors recovered DC by gradient centrifugation, for which they had very heterogeneous populations that could contain variable numbers of cDC and CD163^pos^ cells in every batch including macrophage-like cells.

Recently, other groups have evaluated the susceptibility of *bona fide* cDCs to PRRSV infection, including PRRSV1, PRRSV1.3, and PRRSV2. Resendiz et al. (67) described that tracheal *bona fide* cDCs, specifically cDC1 and cDC2, were not susceptible to PRRSV2 infection but could modulate the expression of cytokines and TLRs. In that study, tracheal explants were incubated with a PRRSV2 strain, and after 24 h of culture, cells with the cDC1 and cCD2 phenotype were sorted. The results showed that cDC1 produced a high level of IFN-α, meanwhile, cDC2 produced IL-10 and up-regulated the expression of TLR2 and TLR4. The authors proposed that cDC1 and cDC2 actively participated in the immunopathology of PRRSV infection (67). In parallel, Bordet et al. (29), demonstrated that *ex vivo* lung cDCs were also refractory to PRRSV1.1 and PRRSV1.3 (Lena strain) infection and that cDC1 and cDC2 had a differential response against PRRSV1.3. In the same study, the authors showed that lung cDC1 from PRRSV 1.3 Lena strain infected animals expressed mRNA of IFN-α, IL-12p35, IL-12p40, and TNF-α, while cDC2 expressed mRNA of the regulatory TGF-β. In contrast, no cytokines were induced by less virulent PRRSV1.1 strains Lelystad or FL13. To note, neither Lena nor the less virulent PRRSV1.1 strains induced an increase in the expression of the maturation markers MHC-I, MHC-II, CD80/86, or CD40.

Recently, Li and Mateu (56), using *in vitro* derived cDCs, also proved the non-susceptibility of cDC1 and cDC2. This work further revealed that cDC1 and cDC2 remained immature upon PRRSV inoculation concerning the expression of maturation molecules, cytokine production, and the endocytosis/phagocytosis capabilities. These results contrast with some previous studies that showed cytokine production after inoculation by PRRSV (29, 68). The differences might be explained by different virus strains used.

**Figure 1:**
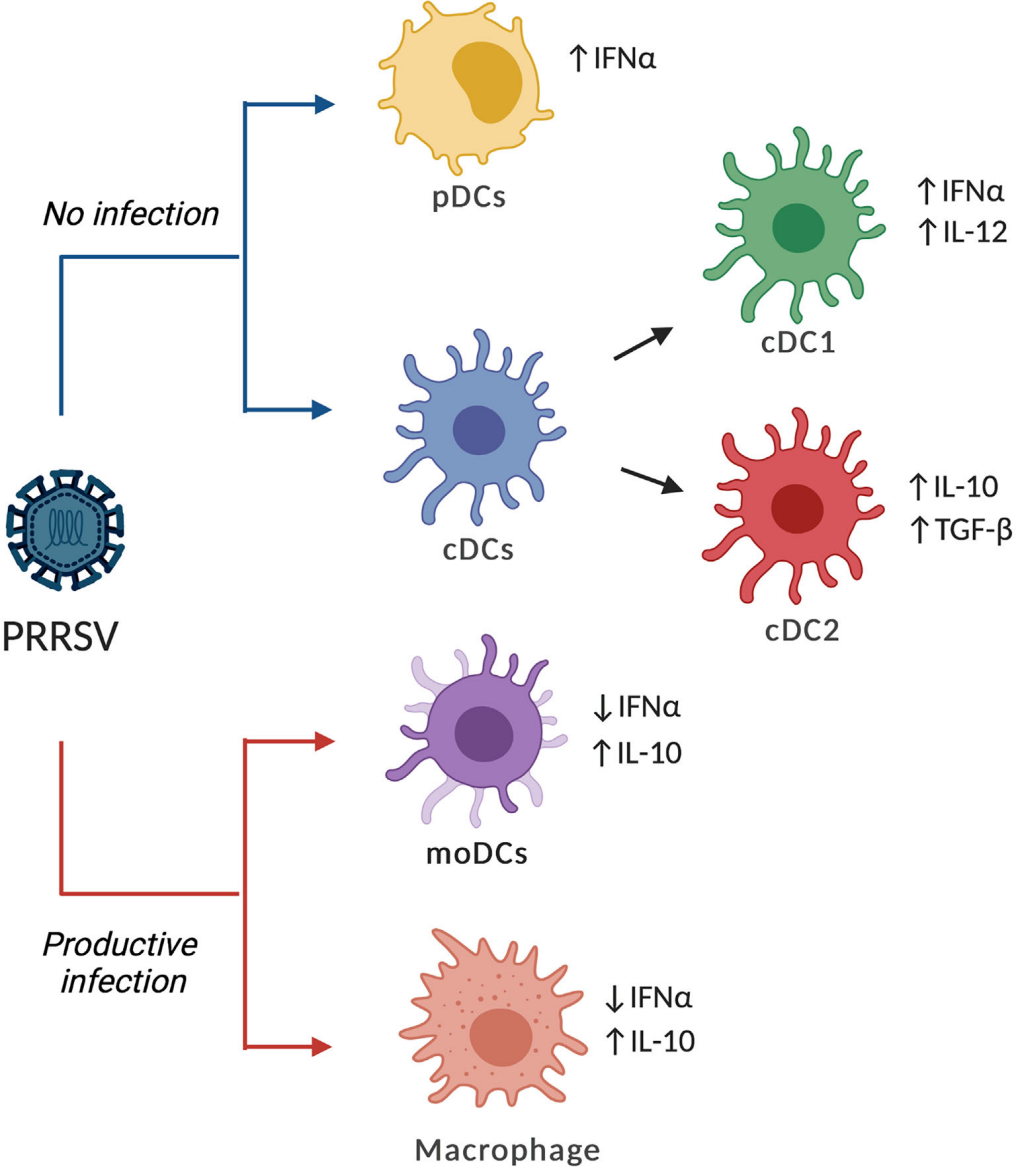
Effects of PRRVS on different types of DCs. PRRSV can infect and replicate on moDCs, bmDCs and macrophages, increasing the expression and production of IL-10 while reducing IFN-α. In contrast, cDCs and pDCs are refractory to PRRSV infection but can respond with the production of cytokines, although the response could be dependent on the strain and the type of DCs. The figure shows the common patterns for PRRSV2 or virulent PRRSV1.3 as reported in the literature.

Puebla-Clark et al. (68), in experimental PRRSV2 infection, showed that at 5- and 7-days post-infection, tonsil cDCs were negative for PRRSV as assessed by detection of PRRSV N protein. Moreover, that study showed that at 5 days after infection cDCs expressed IL-12 mRNA (68). Similar results were observed by Parra-Sánchez et al. (69) with sorted splenic DCs infected with PRRSV2; they showed production of IL-12 evaluated by ELISA (69). In summary, the current studies support the notion that cDCs are not susceptible to PRRSV infection but can differentially respond to the virus. Probably, cDC1 showed a type 1 response and cDC2 showed a type 2 pattern, but this needs to be confirmed in further studies (**Figure 1**). Since the number of cDCs and the ratio of cDC1/cDC2 vary between tissues, the immune response launched in different tissues could be diversified and worth further characterization.

To what extent exposure of cDCs to PRRSV can affect the subsequent T-cell priming and proliferation? Preliminary results by Parra-Sánchez et al. (69) showed that exposure to PRRSV2 did not affect the ability of cDCs to induce proliferation of CD3^+^ T cells, although co-culture with CD3^+^ T cells reduced the production of IL-12 by cDCs (69). In contrast, CD163^+^ cells co-cultured with CD3^+^ T cells in the presence of PRRSV did not affect IL-12 production. It is worth mentioning that cDCs from different tissues could respond differently. For example, blood cDC expressed higher IL-10 levels in comparison to spleen cDCs (69). The number and the ratio of different types of cells could be a reason for such differences. Taken together, cDCs are assumed to poorly participate in launching the adaptive immune response against PRRSV, which, however, remains to be confirmed by further experiments. Interestingly, Li and Mateu (56) reported that sensing of PRRSV by cDC was enhanced when infected cells were used as a source of antigen and hypothesized that the effective sensing of PRRSV by cDCs might not happen at the early stage of infection (56).

It is worth mentioning that virulence of PRRSV strains and interaction with DC seem to be related. Thus, while moderate and low virulence strains might not activate cDCs, strains of higher virulence, such as PRRSV1.3 Lena, induce a stronger Th1 polarization and strongly upregulate several inflammatory, interferon, and apoptosis pathways compared with the less virulent isolates (29, 70). Certainly, the accumulating evidence suggests that this stronger regulation, particularly DCs, may play a role in the immunopathology of those highly virulent strains.

## Plasmacytoid Dendritic Cells Againts PRRSV

pDCs are highly specialized in producing large amounts of type I IFN in response to viral infection. A consensus has been reached that pDCs are not susceptible to PRRSV (71–73). But the production of type I IFN against PRRSV remains controversial. Calzada-Nova et al. (71) indicated that PRRSV did not induce but inhibited IFN-α production in pDCs that were stimulated by the TLR9 ligand. Later, Baumann et al. (72) evaluated different PRRSV1 and PRRSV2 strains and demonstrated that pDCs produced IFN-α in response to PRRSV and that only the highly virulent PRRSV2 strain inhibited IFN-α production (72). In this case, discrepancies were explained by different approaches of separating pDCs.

Exposure of pDCs to PRRSV did not significantly inhibit the natural capability of these cells to produce IFN-α except for some PRRSV2 strains, including the highly pathogenic PRRSV2 that emerged in China in 2006 and the highly virulent PRRSV1.3 Lena strain. When pDCs were exposed to infected macrophages, the IFN-α production was higher than that obtained when using cell-free virions (73), similar to what has been reported recently for cDCs (56). Efficient sensing of infected macrophages by pDCs required integrin-mediated intercellular contact and intact actin filaments in the macrophages (73). The potential contribution of IFN-α to the immunity and immunopathology of PRRSV requires further *in vivo* studies.

## DC Vaccinology: A Strategy to Control PRRSV

Modulation of DC response is a field of continuous exploration, and TLR is a widely studied area (74). The effects of TLR3 and TLR7 ligands were assessed, individually or in combination, for their potential to improve the response of moDCs to an inactivated PRRSV antigen (75). The results suggested that Th1 and Th2 responses were enhanced by TLR3 and TLR7 activation *via* TRIF/MYD88-NF-kB signaling pathway and, therefore, these types of ligands might be new vaccine candidates (75). However, the lack of inclusion of groups where moDCs were stimulated with TLR ligands alone makes difficult to produce a definitive conclusion. In a study by Li and Mateu (56) the authors showed that simultaneous addition of a TLR7 ligand and PRRSV resulted in impaired response, particularly in cytokine production. Therefore, the use of TLR7 ligand as the adjuvant of PRRSV vaccines should be further examined.

DC targeting is a promising approach to stimulate the immune system in mice, humans, and animal species in the veterinary field (76). Recently two works described this strategy to modulate the immune response. Subramaniam et al. (77) evaluated the targeting of three different receptors: DC-SIGN, DEC205, and Langerin, using the ectodomain regions of structural proteins of PRRSV (GP3, GP4, GP5, and M). The authors concluded that this approach did not induce protective immunity as viremia and lung lesions were not reduced in treated groups (77). With a similar approach but using selected peptides from structural and non-structural proteins of PRRSV, Bustamante-Córdova et al. (78) obtained similar results. More studies are needed to identify if this strategy is suitable for PRRSV in combination with the identification of more immunogenic antigens, better immunization routes, and adjuvant combinations.

## Conclusions and Further Research

The current knowledge about PRRSV and its interaction with porcine DCs is still scarce, but some concepts are consolidating. While bmDCs and moDCs may contain subsets of cells that support PRRSV replication (probably because of the expression of the viral receptor CD163), cDCs and pDCs seem not to be susceptible. When the response of cDCs and pDCs was examined against different strains, the results were controversial. Using PRRSV2, cDC1 produced IL-12 and IFN-α, while cDC2 produced IL-10 and TGF-β. Using PRRSV1, low to moderate virulence strains did not induce significant cytokine release in either cDC1 or cDC2 while the highly virulent Lena strain (PRRSV1.3.) induced IL-12-p40 expression in cDC1 and led to a Th1 polarization. Moreover, the distribution of cDC1 and cDC2 in blood, lung and lymphoid tissues is different, and, accordingly, differences can be expected in those tissues. When pDC were examined, only again, the highly virulent PRRSV2 strain inhibited IFN-α release but less virulent strains induced the release of this cytokine. Apart from the current observations, some critical questions remain to be determined. How does the interaction of the virus with DCs interfere with the development of adaptive immune response? In detail, can cDCs and pDCs efficiently capture, process and, present PRRSV antigens? What is the mechanism and what are the receptors involved? Does the exposure to the virus (either live or inactivated) affect the antigen-presenting capabilities of DCs? Resolution of the role of DCs during PRRSV infection would be critical to producing an effective vaccine.

## Author Contributions

JH: conception. JH: writing original draft. YL and EM: writing, reviewing, and editing of the manuscript. All authors contributed to the article and approved the submitted version.

## Funding

This work was supported by the Consejo Nacional de Ciencia y Tecnología (CONACyT), grant number 222973.

## Conflict of Interest

The authors declare that the research was conducted in the absence of any commercial or financial relationships that could be construed as a potential conflict of interest.

## Publisher’s Note

All claims expressed in this article are solely those of the authors and do not necessarily represent those of their affiliated organizations, or those of the publisher, the editors and the reviewers. Any product that may be evaluated in this article, or claim that may be made by its manufacturer, is not guaranteed or endorsed by the publisher.
